# Identifying Potential Clinical Syndromes of Hepatocellular Carcinoma Using PSO-Based Hierarchical Feature Selection Algorithm

**DOI:** 10.1155/2014/127572

**Published:** 2014-03-17

**Authors:** Zhiwei Ji, Bing Wang

**Affiliations:** ^1^School of Electronics and Information Engineering, Tongji University, Shanghai 201804, China; ^2^The Advanced Research Institute of Intelligent Sensing Network, Tongji University, Shanghai 201804, China; ^3^The Key Laboratory of Embedded System and Service Computing, Tongji University, Ministry of Education, Shanghai 201804, China

## Abstract

Hepatocellular carcinoma (HCC) is one of the most common malignant tumors. Clinical symptoms attributable to HCC are usually absent, thus often miss the best therapeutic opportunities. Traditional Chinese Medicine (TCM) plays an active role in diagnosis and treatment of HCC. In this paper, we proposed a particle swarm optimization-based hierarchical feature selection (PSOHFS) model to infer potential syndromes for diagnosis of HCC. Firstly, the hierarchical feature representation is developed by a three-layer tree. The clinical symptoms and positive score of patient are leaf nodes and root in the tree, respectively, while each syndrome feature on the middle layer is extracted from a group of symptoms. Secondly, an improved PSO-based algorithm is applied in a new reduced feature space to search an optimal syndrome subset. Based on the result of feature selection, the causal relationships of symptoms and syndromes are inferred via Bayesian networks. In our experiment, 147 symptoms were aggregated into 27 groups and 27 syndrome features were extracted. The proposed approach discovered 24 syndromes which obviously improved the diagnosis accuracy. Finally, the Bayesian approach was applied to represent the causal relationships both at symptom and syndrome levels. The results show that our computational model can facilitate the clinical diagnosis of HCC.

## 1. Introduction

Hepatocellular carcinoma (HCC) is the third most common cause of cancer-related death worldwide and the leading cause of death in patients with cirrhosis [1, 2]. In clinical practice, symptoms attributable to HCC are usually absent, so the majority of patients are diagnosed with advanced disease, often precluding potentially curative therapies. This has resulted, in part, in a 5-year overall survival rate of 12% and a median survival following diagnosis ranging from 6 to 20 months [3, 4]. Therefore, timely and accurate diagnosis is very important for treatment of HCC. Currently, the modalities employed in the diagnosis of HCC mainly include cross-sectional imaging, biopsy, and serum AFP, which depend on both the size of the lesion and underlying liver function, and some of them are controversial [5, 6].

Traditional Chinese Medicine (TCM) is one of the most popular complementary and alternative medicine modalities. It plays an active role in diagnosis and treatment of HCC in Chinese and East some Asian countries [7, 8]. Different from other diagnostic methods, it is possible to accurately diagnose HCC using inspection, auscultation and olfaction, inquiry, and pulse taking and palpation [8]. In this study, we will work on a TCM clinical dataset, which is observed from 120 HCC patients. Each patient is observed on 147 clinical symptoms and a positive score is evaluated to indicate total positive strength of symptoms. Based on this TCM dataset, we could achieve two aims: (1) screening the potential clinical syndromes for this cancer and (2) inferring the relationships among the potential clinical features via Bayesian network analysis. However, the computational cost will be exceedingly high if the dimensions of the raw dataset are large. Furthermore, the causal relationships between all the features are difficult to infer because high dimensional data sharply increases the complexity of Bayesian network structure learning [9].

**Figure 1:**
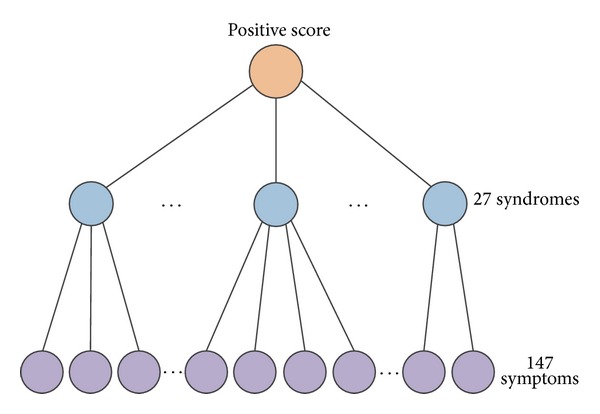
The hierarchical feature representation of TCM clinical dataset.

In this study, a particle swarm optimization-based hierarchical feature selection (PSOHFS) model was proposed to select potential clinical syndromes for HCC diagnoses. Firstly, all the 147 original symptoms were arranged into 27 groups according to the categories of clinical observations, and 27 new syndrome features were generated from these groups. Then, the hierarchical feature representation was built with a tree structure, in which different layers indicate different scales of clinical information (Figure 1). Secondly, an improved PSO algorithm was employed at the syndrome level to search an optimal syndrome subset for diagnoses. The experiment shows that 24 novel syndromes searched by PSOHFS could improve accuracy of diagnosis. In addition, Bayesian networks were further constructed at two levels: (1) a global network on the middle-layer features revealed the relationships among 24 potential syndromes; (2) the local networks were used to represent the connections of symptoms in the same groups.

The rest of the paper is organized as follows.  Section 2 introduces the details about the experimental data and the feature selection approach. Sections 3 and 4 present the experiment design and results, respectively. Some important conclusions drawn are presented in Section 5.

## 2. Materials and Methods

### 2.1. Experimental Data

In this study, the raw data was observed from 120 HCC patients. The clinical dataset includes 300 samples and 147 clinical symptoms. The levels of positive of each symptom are quantified with nonnegative integers. The larger value indicates stronger positive symptom occurred. There are two types of data range for all the original symptoms: binary or integer. For example, the symptom “lip color is white” is binary (0 or 1); that means there are two possible states for this symptom: occurrence or nonoccurrence. Another example is “abdominal pain”; its data range is 0, 1, 2, and 3. The symptom is not positive if its value equals zero; otherwise, the larger the value is, the stronger positive symptom will be. In addition, each patient is marked with a score (nonnegative value) to represent the total evaluation of positive symptoms on this patient. It is obvious that if the HCC patients have larger positive scores than normal people, it is because some clinical symptoms appeared.

### 2.2. Feature Selection

Feature selection for classification or regression can be widely organized into three categories, depending on how they interact with the construction of model. Filter methods employ a criterion to evaluate each feature individually and is independent of the model [10]. Among them, feature ranking is a common method which involves ranking all the features based on a certain measurement and selecting a feature subset which contains high-ranked features [11]. Wrapper methods involve combinatorial searches through the feature space, guided by the predicting performance of a classification or regression model [12]. Embedded methods perform feature selection in the process of training a model [13].

### 2.3. Hierarchical Feature Selection

When the raw dataset is high dimensional, the complexity of feature selection may be extremely high: (a) the computational cost will sharply increase, particularly for the wrapper and embedded methods; (b) the potential optimal feature subset may include many irrelevant or redundant features. Therefore, it is necessary to preliminarily reduce the dimension of original feature set before feature selection. As a common preselecting strategy, feature ranking-based approach could quickly reduce the feature space by picking up high-ranked features [14]. However, this type of approach always leads to inclusion of some redundant features. In addition, the optimal feature subset which covers high-ranked features may not provide the best performance in the classification (or regression) model. Ruvolo et al. proposed a novel hierarchical feature selection approach for the audio classification by converting the raw data to three-layer feature representation with a tree structure [15]. All the low-layer features are aggregated into several groups in a “bag of features” manner, and then a higher-layer feature is extracted based on the lower-layer features in the same group. Obviously, the high-layer feature set constitutes a reduced feature space with little redundancy and might provide lower computational cost for classification or regression model.

In this study, our raw TCM data is high dimensional and there are some redundant clinical symptom features included. For example, there are four redundant observed features to describe lip color of patients, such as “lip color is pale,” “lip color is red,” “lip color is pink,” and “lip color is dark purple.” Therefore, we aggregate several features into a group if they describe the same category of clinical symptoms or the same part of body and define a new syndrome feature for each symptom group. After extracting all the syndrome features, we build a tree structure to achieve the hierarchical feature representation (Figure 1). In this hierarchical structure, the bottom-layer nodes (leaf nodes) are the original clinical symptom features which are directly collected from the original TCM clinical dataset. And a middle-layer syndrome feature is defined on a group of symptoms which are related to the same part of the body. If the symptoms in the same group are not mutually exclusive (concurrent), the corresponding syndrome is defined as the sum of all these symptoms; otherwise, the level of positivity of the syndrome is based on the frequency of each symptom in all the patients (see Section 2). The top-layer node is the root of the tree, which denotes the positive score of a patient. It is obvious that each syndrome roughly represents the positive strength of one specific aspect or part of body, while symptom provides much more detailed information. Particularly, our study focuses on how to reasonably extract the syndrome features to generate a reduced feature set for feature selection and infer the causal relationships among these two-layer features.

### 2.4. Particle Swarm Optimization-Based Hierarchical Feature Selection (PSOHFS)

**Figure 2:**
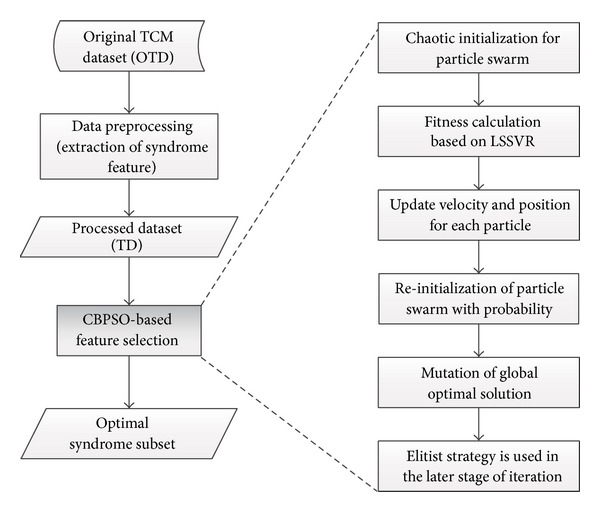
The flow chart of the proposed PSOHFS model for hierarchical feature selection.

Based on the hierarchical feature representation, the dimension of the processed TCM dataset is sharply reduced on the syndrome level. We designed a chaotic binary particle swarm optimization (CBPSO) algorithm to search potential syndromes for diagnosing efficiently. The flow chart of proposed CBPSO-based feature selection is shown in Figure 2.

Particle swarm optimization (PSO) is a population-based random optimization algorithm [16]. A swarm consists of *N* particles moving around in a *D*-dimensional search space. The position of the *i*th particle is represented as *X*_*i*_ = (*x*_*i*1_, *x*_*i*2_,…, *x*_*iD*_), and the velocity *V*_*i*_ = (*v*_*i*1_, *v*_*i*2_,…, *v*_*iD*_), where 1 ≤ *i* ≤ *N*. The positions and velocities of particles are confined within [*X*_min⁡_, *X*_max⁡_]^*D*^ and [*V*_min⁡_, *V*_max⁡_]^*D*^, respectively. Each particle coexists and evolves simultaneously based on knowledge shared with neighboring particles; it makes use of its own memory and knowledge gained by the swarm as a whole to find the best solution. The best previously encountered position of the *i*th particle is considered as its individual best position *p*best_*i*_ = (*p*_*i*1_, *p*_*i*2_,…, *p*_*iD*_). The best position of all the *p*best_*i*_ is considered as the global best position *g*best = (*g*_1_, *g*_2_,…, *g*_*D*_). The limitation of the standard PSO algorithm is applied to optimize the problems in continuous space. However, many optimization problems occur in a discrete feature space; thus binary PSO (BPSO) was proposed to combinatorial optimization [17]. In BPSO, each particle *X*_*i*_ is presented as a binary vector, thus, the overall velocity of particle may be described by the number of bits changed per iteration. Generally, each particle is updated as the following equations:

(1) vidnew=w∗vidold+c1∗r1∗(pbestid−xidold)+c2∗r2∗(gbestd−xidold)if  vidnew∉(Vmin⁡,Vmax⁡),then    vidnew=max⁡⁡(min⁡⁡(Vmax⁡,vidnew),Vmin⁡)S(vidnew)=1(1+e−vidnew)if  rand<S(vidnew),then  xidnew=1;  else  xidnew=0.

Equation (1) will be used to update the velocities and positions of each particle in each generation. The inertia weight *w* controls the impact of the previous velocity of a particle on its current one. *r*_1_ and *r*_2_ are random numbers between [0,1]; *c*_1_ and *c*_2_ are acceleration constants that control how far a particle moves in a single generation. Velocities *v*_*id*_^new^ and *v*_*id*_^old^ denote the *d*th velocities of the *i*th particle in the current and the last generations, respectively. *x*_*id*_^new^ and *x*_*id*_^old^ indicate corresponding positions on the *d*th dimension, respectively. In our case, *V*_max⁡_ = 6, *V*_min⁡_ = −6.

Generally, the speed of convergence of BPSO is fast; however, it has high risk of converging to local optimum. Because chaos is a complex behavior of a nonlinear deterministic system which has ergodic and stochastic properties, we combine chaos theory with BPSO to design chaotic binary particle swarm optimization (CBPSO), which potentially promotes the convergence performance of BPSO [18].

CBPSO-based feature selection is introduced in the following steps (Figure 2).

*(1) Chaotic Initialization of Particle Swarm. *When CBPSO is used for feature selection, each particle indicates a candidate feature subset. Given an original feature set *F* = {*f*_1_, *f*_2_,…, *f*_*D*_}, each particle is denoted by *X*_*i*_ = (*x*_*i*1_, *x*_*i*2_,…, *x*_*iD*_), where *D* is the number of features. It is obvious that each particle represents a candidate feature subset. If *x*_*ij*_ equals 1 indicates the *j*th feature is selected; otherwise, is not selected. The performance of convergence about BPSO largely depends on initial particle swarm. The chaotic initialization via globally searching combined the ergodic and stochastic property of chaotic system is often has a better quality than random initialization.

The common chaotic model is logistic model; it can be shown as follows:

(2) $$\begin{array}{c} q_{k+1}=\mu q_{k}\left(1-q_{k}\right),\;k=0,1,2,\ldots . \end{array}$$

Equation (2) indicates a dynamical system, where *μ* is a control parameter. Given the value of *μ*, a time series *q*_1_, *q*_2_,…, *q*_*k*_ is generated from a random initial value *q*_0_, which ranges from 0 to 1. When *μ* equals 4, there is no stable solution for the dynamic system. It appears as a complete chaotic state.

Now, an initial random vector *X*_0_ = {*x*_01_, *x*_02_,…, *x*_0*D*_} is generated. We substitute each element of *X*_0_ into (2) orderly and iterate *k* times, respectively, and then obtain *D* chaotic variables *CX* = [*x*_1_, *x*_2_,…, *x*_*D*_], which have different locus. When *CX* is substituted into (3), we get *k* binary vectors [*X*_1_; *X*_2_; …; *X*_*k*_], where the binary vector *X*_*j*_ = [*g*(*x*_*j*1_), *g*(*x*_*j*2_),…, *g*(*x*_*jD*_)] represents a particle (1 ≤ *j* ≤ *k*):

(3) $$\begin{array}{c} g\left(x\right)=\{\begin{array}{cc} 1, & x\geq 0.5\\ 0, & x<0.5. \end{array} \end{array}$$

At last, we select *N* top binary vectors to constitute initial particle swarm based on the fitness values. For fully traversal of chaotic variable, the iteration of chaotic series is always large (here, *k* = 500, *N* < *k*). 

*(2) Fitness Calculation Based on LSSVR.* Support vector machine (SVM) has excellent capabilities in classification (SVC) or regression (SVR), even for small sample [19]. It minimizes an upper bound of the generalization error based on the principle of structure risk minimize. However, SVM training process will be time consuming if dataset is huge. Therefore, least squares support vector machine (LSSVM) is proposed to overcome the shortcoming of high computational cost [20]. Generally, LSSVM can be categorized into LSSVR which is used for regression and LSSVC for classification. Because the problem-solving process of the SVR is a QP problem, which will inevitably cause a high computational complexity especially for large-scale QP problem, LSSVR can overcome these shortcomings by a set of linear equations and squared loss function which lead to important reduction in computational complexity [21].

In this study, we use LSSVR as a regression model to evaluate the predicting performance of each candidate feature subset. We assume that an optimal feature subset not only has excellent performance of prediction but also contains more relevant features and less irrelevant features. The fitness function is defined in

(4) $$\begin{array}{c} \text{fitvalue}\;\left(X_{i}\right)=\text{pdterror}\left(X_{i}\right)+p*\text{mfr}\left(X_{i}\right). \end{array}$$

*X*_*i*_ denotes a particle-coding binary vector which indicates a candidate feature subset. The function pdterror(*X*_*i*_) calculates the predicting error of LSSVR model based on the selected features in *X*_*i*_. The parameter *p* is a weight between 0 and 1. Function mfr(*X*_*i*_) indicates the correlation measure between a feature subset and the target variable. In (5), the function fr(*f*_*ij*_) measures the relevance between feature *f*_*ij*_ (included in *X*_*i*_) and target value via a feature-ranking strategy. In our experiment, the more predictive features have smaller values of fr(∗) (see experiment in Section 3.2). Therefore, the smaller fitness value corresponds to the better candidate feature subset:

(5) $$\begin{array}{c} \text{mfr}\left(X_{i}\right)=\text{mean}\left(\text{fr}\left(f_{i1}\right),\text{fr}\left(f_{i2}\right),\ldots ,\text{fr}\left(f_{iM}\right)\right). \end{array}$$

* (3) Update the Velocity and Position for Each Particle.* The velocity and position of each particle are updated according to (1). Considering the searching performance of CBPSO is affected largely by inertia weight (*w*), the value of *w* is dynamically updated in our CBPSO by using nonlinear decreasing strategy. Its calculation is as follows:

(6) $$\begin{array}{c} w=wl*{\left(\frac{ws}{wl}\right)}^{1/(1+c_{3}*(t/\left(t\max\right)))}. \end{array}$$

In (6), *t*max⁡ is the number of iterations, *t* is the current iteration, and *c*_3_ is a constant (set *c*_3_ = 10). *ws* and *wl*, respectively, are the values of *w* on the initial and last generation (*ws* > *wl*). In our case, *ws* = 1.2, *wl* = 0.4. The performance of global search of CBPSO is increased using larger *w* at the beginning of iteration, and the local search will be enhanced using smaller *w* at the later stage.

**Figure 3:**
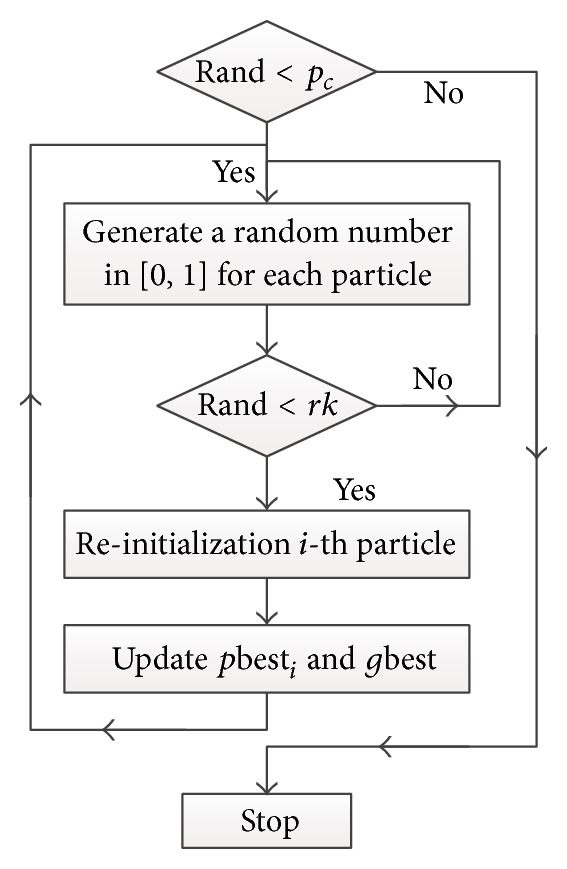
The flow chart of reinitialization of particle swarm.

*(4) Reinitialization of Particle Swarm with Probability.* The trajectory of particle is largely affected by *g*best and all the *p*best_*i*_. At the beginning of iteration, the convergence rate of swarm is fast, but it is slow at the later stage which has high risk of converging to local optimum. For overcoming this shortcoming, each particle in each generation is reinitialized with small probability (Figure 3).

In Figure 3, *p*_*c*_ is the probability of reinitialization for current particle swarm, with its calculation based on (7). At the early stage of iteration, there are many chances for particles to approximate the optimal solution, so that the probability of reinitialization for whole swarm is small. In the later stage, the probability of reinitialization is increased, it can largely avoid the particles fall into the local optimum. The parameter currun denotes the current iteration, and *rk* is a small random probability (in our case, *rk* = 0.3). When the better particle is found after reinitialization, update the current *g*best and *p*best_*i*_:

(7) $$\begin{array}{c} p_{c}=1-\frac{1}{1+\ln\left(\text{currun}\right)}. \end{array}$$

*(5) Mutation of the Potential Global Optimal Solution.* If the global optimal particle *g*best is not constantly improved for a long time, it is necessary to make variation for it to jump out from the local optimal point. In our case, when *g*best is invariant in 10 iterations, its binary coding vector will be mutated with a random probability. If a better particle is found, *g*best is updated again.

*(6) Elitist Strategy Is Used in the Later Stage of Iteration.* If step (4) could not obviously improve the *g*best further, a number of new particles are generated with a probability to instead some particles in current swarm so that the diversity of current swarm could be enhanced [22].

## 3. Experiment

### 3.1. Data Preprocessing

**Figure 4:**
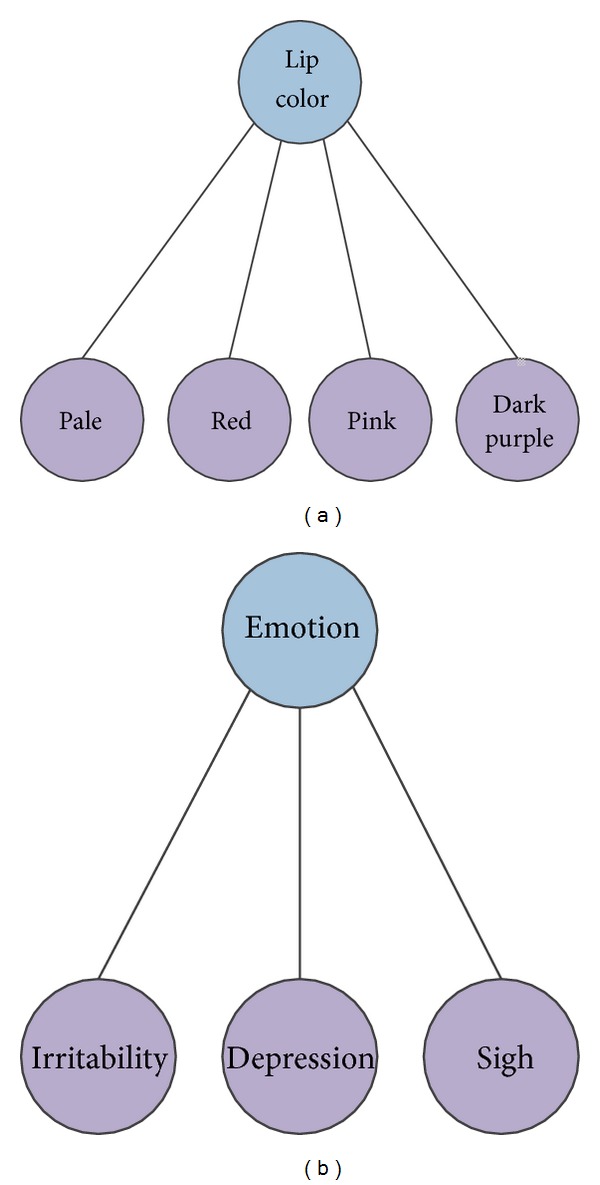
Two groups of symptoms are represented: “lip color” and “emotion.” (a) The syndrome feature “lip color” defined on four clinical symptoms which describe four possible positive states of “lip colors.” (b) The syndrome feature “emotion” defined on three clinical symptom features which describe three types of emotional states.

For hierarchical representation of clinical symptoms, our raw dataset should be preprocessed as in the following steps. Firstly, we manually divide all the 147 symptoms into 27 groups according to the categories of symptoms (Table SS in Supplementary Material available online at http://dx.doi.org/10.1155/2014/127572).  Figure 4(a) shows an example of four clinical symptoms (pale, red, pink, and dark purple) being arranged to a group called “lip color.” Hence, a syndrome feature “lip color” simply represents the states of lip color for a patient instead of four redundant symptom features. Secondly, we calculate each syndrome feature which is extracted from the corresponding clinical symptom group. Therefore, we obtain a new reduced feature space at syndrome level. Finally, combining the original symptom features, extracted syndrome features, and the positive score, we build a tree structure for hierarchical feature representation of the TCM clinical data. Two typical examples are given regarding how to extract the syndrome features from the group of symptoms.

**Example 1**

Figure 4(a) shows an example of several symptoms in the same group being mutually exclusive. That means if the lip color of a patient is red, the rest of the three colors will not appear with him/her. We name a new feature *LC* with five possible discrete values (*LC* = 0,1, 2,3, 4) to simplistically represent the combined meaning of four original symptoms. According to Figure 4(a), the states of lip color for a patient are presented with a binary vector (length is four) in original TCM data, while we can represent it with a single value *LC*, where *LC* ∈ {0,1, 2,3, 4}. If *LC* equals zero, that means all four symptoms are not positive. Otherwise, one of the symptoms appears positive. As for the mapping between four symptoms and four discrete values (1, 2, 3, and 4), we follow a simple rule to assign each candidate value to a possible level of this symptom: the larger discrete value of *LC* indicates that much more patients are positive on this clinical symptom. We count the statistic distributions of all the samples on these four symptoms, respectively, and map each discrete value to a symptom of lip color according to the mean value of positive scores on each symptom.

**Example 2**

The symptoms in the same group are not mutually exclusive.  Figure 4(b) shows three clinical symptoms of emotion: irritability, depression, and sigh. These symptoms could be positive simultaneously on a patient. For example, the clinical symptoms of emotion for a patient are denoted by a vector *Es* = [2,0, 1] in original data, which means two emotion-related positive symptoms appeared with him/her. In this case, a new syndrome feature *N* *Es* is extracted from *Es*, where *N* *Es* = sum(*Es*) = 3. Therefore, if a patient has several positive symptoms which belong to the same syndrome, cumulative summation is a feasible strategy to get a total positive strength on this syndrome.

### 3.2. Experiment Design

First, we proposed a feature-ranking strategy for association analysis between individual syndrome and positive score (target value) with function fr(∗):

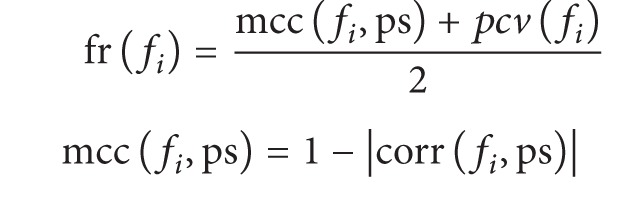
(8)

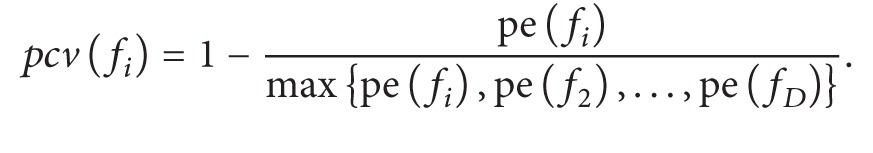
(9)
Combining ((4)-(5), (8)-(9)), we can determine the fitness function in the proposed PSOHFS model for feature subset optimizing. The function corr(*f*_*i*_, ps) is the correlation coefficient between feature *f*_*i*_ and target value (ps). Function pe(*f*_*i*_) denotes the predicting error of LSSVR model with all the features except *f*_*i*_. If the predicting error is obviously increased after moving out *f*_*i*_ from the whole feature set, it indicates the feature *f*_*i*_ is high predictive. The smaller value of fr(*f*_*i*_), the higher-ranked feature *f*_*i*_ will be. The result of feature ranking can provide a reference about the importance of each syndrome to positive score.

Secondly, our developed CBPSO algorithm was applied at the syndrome level for feature selection. Different swarm size and the number of iterations were chosen to test the searching performance of the proposed CBPSO. And then, the predicting performance of the optimal syndrome subset (OPS) by proposed model was further validated. On the one hand, we employed two well-established feature selection methods to compare them with our proposed PSOHFS model: (1) correlation-based filter method (CFM) [14, 23] and (2) PSO-based wrapper method (PWM) [14]. These standard approaches were applied on original symptom features. On the other hand, we further validated the performance of OPS by feature ranking on the syndrome feature level. Two types of syndrome subsets were selected to compare: (1) full collection with all the 27 syndromes (FCS) and (2) filter-based syndrome set by feature ranking via (8). Here, we set threshold 0.8 and 0.9 to get two potential syndrome subsets: FRS1 and FRS2.

Finally, based on the optimal potential syndrome subset inferred by our PSOHFS model, Bayesian networks were constructed, respectively, at the symptom and syndrome levels. On the one hand, the global Bayesian network on potential syndromes was inferred using GES algorithm [24]. Such coarser-grained network can roughly reveal the causal relationships among these potential syndromes of this cancer. Before structure learning of global network, the processed TCM dataset (TD) in Section 3.1 should be firstly discretized according to

(10) DTD(:,j)={TD(:,j),  if  length(unique(TD(:,j)))≤4TD(:,j)max⁡⁡(TD(:,j))/itvnum(TD(:,j)),  elseitvnum(TD(:,j))  =⌊log⁡2(length(unique(TD(:,j))))⌋+1.

TD(:, *j*) denotes all the calculated values of *j*th syndrome. Function itvnum  (*TD*(:, *j*)) is used to estimate the optimal intervals of discretization for the sample of *j*th syndrome. If the number of positive levels for a syndrome is larger than four, the discretization is necessary on this syndrome. On the other hand, we chose three syndromes as examples to construct local networks using GES algorithm (Table 4). When a network structure is learned, Maximum Likelihood Estimation (MLE) is utilized to compute all the conditional probability tables. Then, the probability inference could be achieved using inference algorithm, such as junction tree method [25, 26].

### 3.3. Experimental Parameters

The simulating experiments were implemented under the environment of MATLAB2011a with Intel Core i5-2410 CPU @ 2.3GHZ, 4 GB RAM. In the LSSVR regression model, Gaussian RBF kernel is employed, and the kernel parameters *σ* ^2^ and *γ* should be determined firstly. Currently, many approaches have been applied in parameter optimization of LSSVR, such as grid search [27], cross-validation [28, 29], genetic algorithm (GA) [30], and simulated annealing algorithm [31]. In our study, grid search was selected to determine the parameters in the range of [0.1, 100000] for *σ* ^2^ and [0.1, 10000] for *γ*. For a pairwise (*σ* ^2^, *γ*), we used 10-fold cross-validation to evaluate the performance of LSSVR model.

To evaluate the accuracy of prediction, three statistical metrics are widely employed: (1) mean square error (MSE), (2) root mean square error (RMSE), and (3) mean relative percentage error (MRPE). In (11), where *y*_*i*_ and *y*_*i*_′ are the observed value and predicted value, the smaller MSE, RMSE, and MRPE are, the better the LSSVR model will be:

(11) $$\begin{array}{c} \text{MSE}=\frac{1}{n}\overset{n}{\underset{i=1}{\sum }}{\left[y_{i}-y_{i}^{\prime }\right]}^{2}\\ \text{RMSE}=\sqrt{\frac{1}{n}\overset{n}{\underset{i=1}{\sum }}{\left[y_{i}-y_{i}^{\prime }\right]}^{2}}\\ \text{MRPE}=\frac{1}{n}\overset{n}{\underset{i=1}{\sum }}\left|\frac{y_{i}^{\prime }-y_{i}}{y_{i}}\right|\times 100\%. \end{array}$$

In our experiment, we used MSE to calculate the values of function pdterror(∗) and pe(∗).

Moreover, the Matlab Bayes Net Toolbox FullBNT-1.0.7 [32] and BNT Structure Learning Package BNT_SLP_1.5 were, respectively, used in the Bayesian network structure learning, parameters learning, and probability inference. The probability distribution between nodes in a Bayesian network could be computed according to the inferred network structure and conditional probability tables.

## 4. Results and Discussion

Table 1 shows the results of association analysis between individual syndromes and positive score. mcc(*f*_*i*_, ps) reflects the predicting performance of feature *f*_*i*_ to ps (positive score). The smaller the value of mcc is, the more important the feature *f*_*i*_ will be. The value of pe(*f*_*i*_) indicates predicting error of LSSVR model based on all the features except *f*_*i*_; it is measured by MSE. Here, it is obvious that the higher-ranked features have lower values of fr(*f*_*i*_). We clearly see some important syndromes are high predictive, such as “facial features,” “skin of the limbs,” “diet,” “sternocostal and abdominal pain,” and so forth.

**Figure 5:**
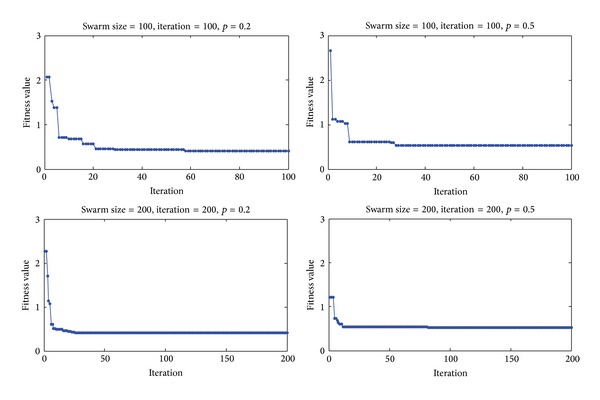
The results of CBPSO-based feature selection under different parameters. Four subfigures show the CBPSO algorithm rapidly approximate the optimal solution in the reduced feature space.

Our developed CBPSO algorithm was applied to search the optimal syndrome subset on the processed TCM dataset. Assigning different swarm size and the number of iterations, this CBPSO algorithm shows excellent convergence performance (Figure 5). Different assignments of parameters for CBPSO finally got the same optimal solution: 001101111111111111111111111. It means the potential syndrome subset containing 24 syndromes is a steady solution for this NP-hard problem (Table 2). These 24 syndromes reflect many cancer-related parts of body or aspects of observation, which are helpful to clinically diagnose HCC.

Now, two well-established feature selection methods were introduced to be compared with our proposed PSOHFS model: (1) correlation-based filter method (CFM) [14, 23] and (2) PSO-based wrapper method (PWM) [14]. The first one uses correlation-based feature ranking as the principle criteria for feature selection by ordering. The second one uses standard BPSO algorithm to search an optimal feature subset. These two methods were all applied on the original symptom features. For CFM, we used 15% and 30% top-ranked features to validate its performance, while, for PWM, we set population size equal to 100 and iterations equal to 100 and 200.  Table 3 shows the error of prediction of the LSSVR model based on candidate optimal feature subsets. Five candidate feature subsets were searched by the above two methods and PSOHFS model, respectively. In Table 3, the values of MSE, MRSE, and MRPE were calculated based on LSSVR by 5-fold cross-validation.

Comparing the values of MSE, RMSE, and MRPE in Table 3, we can see that the optimal syndrome set (OPS) searched by our PSOHFS model has the obvious superiority in the predicting performance. The dimension of the PSOHFS-based optimal syndrome subset equals 24, which is significantly smaller relatively to the dimension of the original symptoms (147). Because CFM and PWM work directly on the original high dimensional feature space, it is hard for them to achieve an optimized prediction performance and the dimension of potential feature subset, simultaneously. PWM searches for the optimal solution depending on the evaluation of regression model, so the optimal feature subset from PWM is more predictive than CFM's. However, standard wrapper-based methods do not optimize the size of optimal feature subset. CFM got the worst result is reasonable because the correlation measurement can only detect linear dependencies between variable and target.

Next, we further validate the performance of OPS on the syndrome level. Two types of syndrome subsets were selected to compare: (1) full collection with all the 27 syndromes (FCS) and (2) filter-based syndrome subset by feature ranking via (8). Here, we chose threshold 0.8 and 0.9 to get two potential syndrome subsets: FRS1 and FRS2 (Table 1). In Table 4, we obviously find OPS can get good balance between the dimension and predicting performance. The verification on FRS1 and FRS2 proves the fact that, although feature-ranking methods run quickly, they still easily lead to worse results because feature-ranking filter ignores the possible interactions and dependences among the features [29]. The difference between Tables 3 and 4 indicates the feature selection on a reduced feature space of original dataset potentially obtains a better solution. 24 potential syndrome features could quickly diagnose the positive level of HCC patients with high accuracy. Our result suggested that “lip color,” “tongue color,” and “coated tongue color” could be ignored during the process of prediction because they are weak predictive features for discriminating these HCC samples.

**Figure 6:**
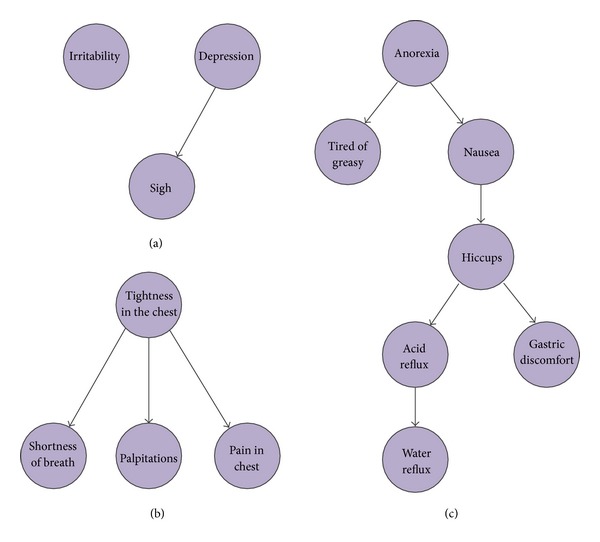
Three inferred Bayesian networks based on symptom features. (a) The casual relationships among three clinical symptoms of “emotion” group. “Depression” might cause “sigh,” while “irritability” is an isolated node. (b) The casual relationships among four clinical symptoms of “cardiothoracic condition” group. (c) The casual relationships among seven clinical symptoms of “diet.”

**Figure 7:**
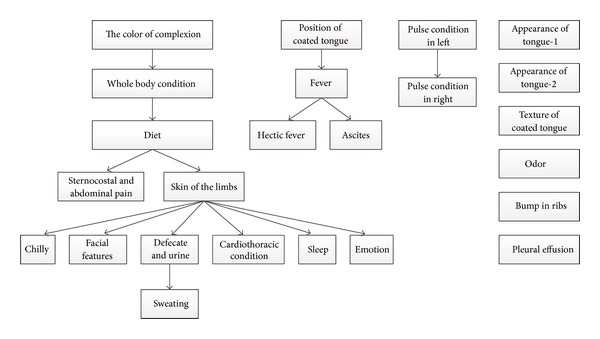
The global Bayesian network based on 24 potential syndromes.

Finally, based on the hierarchical feature representation and the result of feature selection on syndromes, Bayesian network on two layers was constructed and the conditional probability tables were inferred. Here, we picked up three cases to explain what we can obtain from the Bayesian network analysis in the symptom and syndrome feature space (Table 5).  Figure 6(a) shows the Bayesian network structure of “emotion” syndrome. We can clearly see that there is a causal relationship between “depression” and “sigh.” When a patient is depressive, sigh is a usual symptom with him/her. While “irritability” seems to reflect inversely comparing to “depression”; therefore it is an independent node in this inferred network structure. The conditional probability tables of “emotion” are shown as in Supplementary Table S1A-S1C. For example, P(“irritability” = 0, “depression” = 1, “sigh” = 1) = 0.027 suggests the probability of the clinical symptoms “depression” and “sigh” is positive on a patient.  Figure 6(b) shows the network structure of “cardiothoracic condition” syndrome. From Figure 6(b), “tightness in the chest” might lead to three other clinical symptoms: “shortness of breath,” “palpitations,” and “pain in chest.” The conditional probability tables of “cardiothoracic condition” are shown in Supplementary Table S2A-S2D. For example, P(“tightness in the chest” = 1, “shortness of breath” = 1, “palpitations” = 1, “pain in chest” = 0) = 0.01143. Similarly, Figure 6(c) shows the network structure of “diet” syndrome. The conditional probability tables of “diet” are shown in Supplementary Table S3A-S3G. At last, Figure 7 represents the global network on 24 potential syndromes. There are three subnetwork modules and six independent nodes in Figure 7. All the relationships among these syndromes were represented. Their conditional probability tables were listed in Supplementary Table SS1-SS24. Based on the hierarchical feature representation, the Bayesian networks potentially provided us with useful knowledge with multi-granularity. From Table 6, we can clearly see that the computational cost of network structure learning is sharply increased when the number of nodes in the network is increasing. It further proves that if we construct Bayesian network on 147 original clinical symptoms directly, it will meet unimaginable computational complex; therefore, our method proposed in this paper provided a good solution.

## 5. Conclusions

In this paper, a particle swarm optimization-based hierarchical feature selection (PSOHFS) model was proposed to infer potential clinical features of HCC on a Traditional Chinese Medicine dataset which was collected from 120 patients. The PSOHFS model firstly arranged all the 147 original symptoms into 27 groups according to the categories of clinical symptoms and extracted a new syndrome feature from each group. The raw TCM clinical dataset was represented in a reduced feature space so that we can build a hierarchical feature representation pattern with a tree structure. Based on such hierarchical feature graph, we reached two aims: (1) based on a significant reduced feature space, the feature selection can be easily realized, and the optimal feature subset could diagnose patient samples efficiently; (2) we constructed Bayesian network on symptom and syndrome levels. A global Bayesian network for all the potential syndromes roughly described the relationships among the main important aspects of HCC. While each local network was constructed for the symptom features in the same group, the causal relationships among them could be inferred.

In our simulating experiment, our CBPSO algorithm in PSOHFS model discovered an optimal syndrome subset of HCC, which included 24 syndromes. With a LSSVR regression model built by these 24 potential syndromes, the diagnosis accuracy of HCC is high and computational cost is sharply reduced. The significance of the proposed model is as follows: (1) feature selection is implemented on a reduced feature space, so that the dimension of optimal feature subset is smaller; (2) the fitness function in CBPSO algorithm optimizes the predicting performance and the correlation between features and target variable. Based on the results of feature selection, we further achieved the Bayesian network construction at both syndrome and symptom levels to explain the relationships among all the nodes and the probability inference could be computed based on learned network structure and conditional probability tables.

However, our model also has some shortcomings: (1) most of syndrome groups were aggregated from the clinical symptoms observed from the same parts of body, while much more evidence proved that there are significant relationships between symptoms which describe different parts (aspects) of body; (2) we did not study the relationships of clinical symptom features which belong to different groups. In the future, we will collect more clinical samples of HCC to deeply analyze the correlation between any clinical features. Also, some high-predictive clinical features inferred in this study need to be validated further in other TCM clinical datasets. If we can discover and validate some high-predictive clinical features in the next step of research, that might be the significant phenotypes of this cancer.

## Supplementary Material

Supplementary Materials: In this section, the detailed information of all the symptoms and syndromes were firstly described. In Table SS, each syndrome group and the symptoms which were arranged into the same group were presented. Table S1A-S1C, S2A-S2D, and S3A-S3G show the conditional probability tables of the Bayesian network "Emotion", "Cardiothoracic condition", and "Diet", respectively (Figure 6). As to the global network of all the 24 potential syndromes (Figure 7), all the conditional probability tables were presented in Table SS1-SS24.

## Figures and Tables

**Table 1:** The result of feature ranking for all the syndromes.

Syndrome (*f*_*i*_)	Name of syndrome	Abbreviation	Size	mcc(*f*_*i*_, *ps*)	pe(*f*_*i*_)	fr(*f*_*i*_)	Rank
1	Lip color	LC	4	0.9257	0.1688	0.9480	24
2	Tongue color	Tc	4	0.9293	0.1813	0.9487	25
3	Appearance of tongue-1	At1	3	0.8123	0.5808	0.8550	16
4	Appearance of tongue-2	At2	5	0.9712	0.2998	0.9592	27
5	Coated tongue color	Ctc	3	0.8589	0.1914	0.9126	21
6	Texture of coated tongue	Tct	7	0.9039	0.2518	0.9298	23
7	Position of coated tongue	Pct	5	0.9629	0.2685	0.9578	26
8	The color of complexion	Coc	8	0.6396	2.7350	0.5790	6
9	Whole body condition	Wbc	8	0.9326	1.0378	0.8749	19
10	Odor	Od	1	0.6948	0.6055	0.7941	13
11	Chilly	Ch	1	0.6011	0.4767	0.7586	10
12	Hectic fever	Hf	1	0.7890	0.4248	0.8571	17
13	Fever	Fe	1	0.7304	0.2969	0.8391	15
14	Sweating	St	2	0.6270	0.4875	0.7706	11
15	Facial features	Ff	13	0.2177	5.6792	0.1088	1
16	Cardiothoracic condition	Ca	4	0.4923	1.2036	0.6402	8
17	Sternocostal and abdominal pain	Sap	16	0.4010	1.6943	0.5513	5
18	Diet	Diet	7	0.2937	1.7266	0.4948	3
19	Defecate and urine	Du	10	0.4016	1.7268	0.5488	4
20	Sleep	Slp	2	0.4382	0.9854	0.6324	7
21	Emotion	NEs	3	0.5141	1.1600	0.6549	9
22	Skin of the limbs	Sl	10	0.2091	2.4312	0.3905	2
23	Bump in ribs	Bir	1	0.6543	0.5541	0.7784	12
24	Ascites	Ass	1	0.7279	0.4304	0.8260	14
25	Pleural effusion	Pe	1	0.7894	0.2301	0.8745	18
26	Pulse condition in left	Pcle	13	0.8630	0.3003	0.9051	20
27	Pulse condition in right	Pcrt	13	0.8716	0.2556	0.9133	22

**Table 2:** The optimal solutions of our CBPSO using different parameters.

Swarm size	Iteration	*P*	The optimal solution of CBPSO	Fitness value
100	100	0.2	001101111111111111111111111	0.40911
100	100	0.5	001101111111111111111111111	0.53205
200	200	0.2	001101111111111111111111111	0.41062
200	200	0.5	001101111111111111111111111	0.52183

**Table 3:** The predicting performance of the optimal feature subsets obtained from different feature selection methods.

Approaches	Dimension of the optimal feature subset	MSE	RMSE	MRPE (%)	Time (second)
PSOHFS	**24 (syndromes)**	**0.1622**	**0.4027**	**1.0700**	**3.0108**
CFM (top 15%)	22 (symptoms)	14.4575	3.8023	11.8907	2.8510
CFM (top 30%)	45 (symptoms)	6.2611	2.5022	7.8632	4.8010
PWM (100 iterations)	92 (symptoms)	3.2268	1.7963	5.5645	8.8760
PWM (200 iterations)	89 (symptoms)	2.7516	1.6588	5.2351	8.7390

**Table 4:** Comparisons of the PSOHFS-based optimal syndrome set with other potential syndrome subsets.

Feature set	Dimension	MSE	RMSE	MRPE (%)	Time (second)
OPS	**24**	**0.1622**	**0.4027**	**1.0700**	**3.0108**
FCS	27	0.1834	0.4283	1.9572	3.2604
FRS1	13	3.3735	1.8367	6.2871	2.4024
FRS2	19	1.7084	1.3071	4.5202	2.9640

**Table 5:** The details of three syndromes.

Syndrome	Symptoms	The number of level of positive symptom
Emotion	Irritability	4
	Depression	3
	Sigh	3

Cardiothoracic condition	Tightness in the chest	4
	Shortness of breath	3
	Palpitations	3
	Pain in the chest	3

Diet	Anorexia	4
	Tired of greasy	4
	Nausea	3
	Hiccups	3
	Acid reflux	3
	Water reflux	3
	Gastric discomfort	2

**Table 6:** The computational cost of structure learning for some Bayesian networks.

Bayesian networks	The number of nodes	Computational time of structure learning (second)
Emotion	3	0.06
Cardiothoracic condition	4	0.41
Diet	7	4.23
Potential syndrome set (OPS)	24	606.88
