# Supplementary material for: Identifying Potential Clinical Syndromes of Hepatocellular Carcinoma Using PSO-Based Hierarchical Feature Selection Algorithm
Source: Biomed Res Int. 2014 Mar 17;2014:127572. doi: 10.1155/2014/127572 (PMC3976846; doi:10.1155/2014/127572)
Supplement: Supplementary file 1 — Supplementary Materials: In this section, the detailed information of all the symptoms and syndromes were firstly described. In Table SS, each syndrome group and the symptoms which were arranged into the same group were presented. Table S1A-S1C, S2A-S2D, and S3A-S3G show the conditional probability tables of the Bayesian network "Emotion", "Cardiothoracic condition", and "Diet", respectively (Figure 6). As to the global network of all the 24 potential syndromes (Figure 7), all the conditional probability tables were presented in Table SS1-SS24. [file 127572.f1.pdf]

Identifying potential clinical syndromes of Hepatocellular carcinoma Using PSO-based hierarchical feature selection algorithm

Zhiwei Ji [1], Bing Wang [1,2,3*]

[1]School of Electronics and Information Engineering, Tongji University, Shanghai, 201804, China

[2]The Advanced Research Institute of Intelligent Sensing Network, Tongji University, Shanghai, 201804, China

[3]The Key Laboratory of Embedded System and Service Computing, Ministry of Education, Tongji University, Shanghai, 201804, China

Supplementary Materials

Table SS: The name list of all the symptoms and syndromes.

ID.	Syndromes	Symptoms (by categories)
1	Lip color	Pale, Red, Pink, Dark purple
2	Tongue color	Pale, Red, Deep red, Deep blue hue
3	Appearance of tongue-1	Enlarged, Teeth-printed, Petechial
4	Appearance of tongue-2	Cracked, Prick in tongue tip, Prick in margin of the tongue, Smooth, Cirsoid vein in hypoglossis
5	Coated tongue color	White, Yellow, White and Yellow
6	Texture of coated tongue	Thick tongue, Thin tongue, Greasy tongue, Dry tongue, Slippery tongue, No tongue, Peeling tongue
7	Position of coated tongue	Tongue tip, Left side of tongue , Right side of tongue, Root of tongue, Overall tongue
8	The color of complexion	Sallow complexion, Yellow complexion , Pale complexion, Gloomy complexion, Flushing complexion, Black brown complexion, Red complexion, Hectic cheek
9	Whole body condition	Palmar Erythema, Spider Angioma, Prominent veins, Dropsical legs, Encrusted skin, Black eye socket, Sclera ecchymosis, Puffy face
10	Odor	Dirty mouth
11	Chilly	Chilly
12	Hectic fever	Hectic fever
13	Fever	Fever
14	Sweating	Sweating , Sweating at night
15	Facial features	Dizziness, Dry eye, Tinnitus, Amnesia, Tastelessness, Bitter taste, Xerostomia,

		Greasy in mouth, Thirst and like drinking, Thirst and dislike drinking, Bleeding, Blurred vision, Tongue sore
16	Cardiothoracic	Tightness in the chest, Shortness of breath, Palpitations, Pain in chest
17	Sternocostal and abdominal pain	Pain in rib, Swelling pain-1, Stabbing pain-1, Dull pain-1, Swelling pain-2, Stabbing pain-2, Dull pain-2, Stomachache, Pain in uncertain place, Prefer warmth, Prefer cool, Burning pain, Pleasure-relieved pain, Pain in a place, fullness and distention in stomach, fullness and distention in abdomen
18	Diet	Anorexia, Tired of greasy, Nausea, Hiccups, Acid reflux, Gastric discomfort, Water reflux
19	Defecate and urine	Dry stool, Unwell defecate, Yellow urine, Frequent urine at night, less urine, Retention of urine, Frequent and limpid urine, Bowel vector gas, loose stool, Semiliquid stool
20	Sleep	Sleeplessness, Dreaminess
21	Emotion	Irritability, Depression, Sigh
22	Skin of the limbs	Limp waist-leg, Deadlimb, Five frustrating heat, Cold hands and feet, Tired and weak, Heaviness of limbs, Skin itch, Arthralgia, Crymodynia in joint, Heat pain in joint
23	Bump in ribs	Bump in ribs
24	Ascites	Ascites
25	Pleural effusion	Pleural effusion
26	Pulse condition in left	Floating pulse, Full pulse, Deep pulse, Soft pulse, Slow pulse, Moderate pulse, Stringy pulse, Weak pulse, Slippery pulse, Rapid pulse, Uneven pulse, Thready pulse, Irregular pulse
27	Pulse condition in right	Floating pulse, Full pulse, Deep pulse, Soft pulse, Slow pulse, Moderate pulse, Stringy pulse, Weak pulse, Slippery pulse, Rapid pulse, Uneven pulse, Thready pulse, Irregular pulse

Table S1A: The Conditional Probability Table of “Irritability ”.

P(Irritability =0)	P(Irritability =1)	P(Irritability =2)	P(Irritability =3)
0.7367	0.1767	0.08	0.0067

Table S1B: The Conditional Probability Table of “Depression”.

P(Depression =0)	P(Depression =1)	P(Depression =2)
0.8	0.17	0.03

Table S1C: The Conditional Probability Table of “Sigh”.

	P(Sigh=0)	P(Sigh=1)	P(Sigh=2)
Depression =0	0.9375	0.0583	0.0042
Depression =1	0.7647	0.2157	0.0196
Depression =2	0.6667	0	0.3333

Table S2A: The Conditional Probability Table of “Tightness in the chest (TITC)”.

P(TITC=0)	P(TITC=1)	P(TITC=2)	P(TITC=3)
0.8433	0.1267	0.0267	0.0033

Table S2B: The Conditional Probability Table of “Shortness of breath (SOB)”.

	P(SOB=0)	P(SOB=1)	P(SOB=2)
TITC=0	0.9407	0.0435	0.0158
TITC=1	0.5789	0.3947	0.0263
TITC=2	0.25	0.125	0.625
TITC=3	0	1	0

Table S2C: The Conditional Probability Table of “Palpitations (Ps)”.

	P(Ps=0)	P(Ps=1)	P(Ps=2)
TITC=0	0.9486	0.0395	0.0119
TITC=1	0.6842	0.2895	0.0263
TITC=2	0.375	0.625	0
TITC=3	1	0	0

Table S2D: The Conditional Probability Table of “Pain in chest (PIC)”.

	P(PIC=0)	P(PIC=1)	P(PIC=2)
TITC=0	0.9802	0.0158	0.004
TITC=1	0.7895	0.1842	0.0263
TITC=2	0.75	0.25	0
TITC=3	1	0	0

Table S3A: The Conditional Probability Table of “Anorexia”.

P(Anorexia =0)	P(Anorexia =1)	P(Anorexia =2)	P(Anorexia =3)
0.7633	0.1833	0.0467	0.0067

Table S3B: The Conditional Probability Table of “Tired of greasy (TOG)”.

	P(TOG=0)	P(TOG=1)	P(TOG=2)	P(TOG=3)
Anorexia =0	0.8734	0.0961	0.0306	0
Anorexia =1	0.6727	0.2727	0.0545	0
Anorexia =2	0.2143	0.4286	0.3571	0
Anorexia =3	0.5	0	0	0.5

Table S3C: The Conditional Probability Table of “Nausea”.

	P(Nausea =0)	P(Nausea =1)	P(Nausea =2)
Anorexia =0	0.9258	0.0699	0.0044
Anorexia =1	0.6727	0.2909	0.0364
Anorexia =2	0.8571	0	0.1429
Anorexia =3	1	0	0

Table S3D: The Conditional Probability Table of “Hiccups”.

	P(Hiccups =0)	P(Hiccups =1)	P(Hiccups =2)
Nausea =0	0.943	0.0494	0.0076
Nausea =1	0.7188	0.25	0.0313
Nausea =2	0.4	0.2	0.4

Table S3E: The Conditional Probability Table of “Acid reflux (AR)”.

	P(AR=0)	P(AR =1)	P(AR =2)
Hiccups =0	0.9377	0.0586	0.0037
Hiccups =1	0.5455	0.4091	0.0455
Hiccups =2	0.6	0.4	0

Table S3F: The Conditional Probability Table of “Water reflux (WR)”.

	P(WR=0)	P(WR =1)	P(WR =2)
AR =0	0.9377	0.0037	0
AR =1	0.8519	0.1481	0
AR =2	0	0.5	0.5

Table S3G: The Conditional Probability Table of “Gastric discomfort (GCDT)”.

	P(GCDT=0)	P(GCDT =1)
Hiccups =0	0.9634	0.0366
Hiccups =1	0.6364	0.3636
Hiccups =2	0.8	0.2

Table SS1: The Conditional Probability Table of “Appearance of tongue 1 (At1)”.

P(At1 =0)	P(At1=1)	P(At1=2)
0.4267	0.4867	0.0867

Table SS2: The Conditional Probability Table of “Appearance of tongue 2 (At2)”.

P(At2 =0)	P(At2=1)	P(At2=2)
0.9033	0.0367	0.06

Table SS3: The Conditional Probability Table of “Texture of coated tongue (Tct)”.

P(Tct =0)	P(Tct=1)	P(Tct=2)	P(Tct=3)
0.0677	0.2833	0.63	0.02

Table SS4: The Conditional Probability Table of “Odor (Od)”.

P(Od=0)	P(Od=1)	P(Od=2)	P(Od=3)
0.8133	0.1133	0.0633	0.01

Table SS5: The Conditional Probability Table of “Bump in ribs (Bir)”.

P(Bir =0)	P(Bir=1)	P(Bir=2)	P(Bir=3)
0.7733	0.163	0.0333	0.03

Table SS6: The Conditional Probability Table of “Pleural effusion (Pe)”.

P(Pe =0)	P(Pe=1)	P(Pe=2)
0.9733	0.0133	0.0133

Table SS7: The Conditional Probability Table of “Pulse condition in left (Pcle)”.

P(Pcle =0)	P(Pcle=1)	P(Pcle=2)	P(Pcle=3)
0.02	0.5567	0.4067	0.0167

Table SS8: The Conditional Probability Table of “Pulse condition in right (Pcrt)”.

	P(Pcrt=0)	P(Pcrt=1)	P(Pcrt=2)	P(Pcrt=3)
Pcle =0	1	0	0	0
Pcle =1	0	0.9701	0.0299	0
Pcle =2	0.0082	0.1393	0.8361	0.0164
Pcle =3	0	0	0	1

Table SS9: The Conditional Probability Table of “Position of coated tongue (Pct)”.

P(Pct =0)	P(Pct=1)	P(Pct=2)
0.7567	0.2367	0.0067

Table SS10: The Conditional Probability Table of “Fever (Fe)”.

	P(Fe=0)	P(Fe=1)	P(Fe=2)
Pct =0	0.9515	0.022	0.0264
Pct =1	0.9014	0.0986	0
Pct =2	0.5	0.5	0

Table SS11: The Conditional Probability Table of “Hectic fever (Hf)”.

	P(Hf=0)	P(Hf=1)	P(Hf=2)	P(Hf=3)
Fe =0	0.9786	0.0142	0.0071	0
Fe =1	0.5385	0.0769	0.3846	0
Fe =2	0.8333	0	0	0.1667

Table SS12: The Conditional Probability Table of “Ascites (Ass)”.

	P(Ass=0)	P(Ass=1)	P(Ass=2)	P(Ass=3)
Fe =0	0.9217	0.0569	0.0142	0.0071
Fe =1	0.6154	0	0.3077	0.0769
Fe =2	0.3333	0.3333	0.3333	0

Table SS13: The Conditional Probability Table of “The color of complexion (Coc)”.

P(Coc =0)	P(Coc=1)	P(Coc=2)	P(Coc=3)
0.58	0.3367	0.0567	0.0267

Table SS14: The Conditional Probability Table of “Whole body condition (Wbc)”.

	P(Wbc=0)	P(Wbc=1)	P(Wbc=2)
Coc =0	0.7184	0.2816	0
Coc =1	0.5545	0.3762	0.0693
Coc =2	0.4118	0.4706	0.1176
Coc =3	0.125	0.625	0.25

Table SS15: The Conditional Probability Table of “Diet”

	P(Diet=0)	P(Diet=1)	P(Diet=2)	P(Diet=3)
Wbc =0	0.8995	0.1005	0	0
Wbc =1	0.79	0.14	0.05	0.02
Wbc =2	1	0	0	0

Table SS16: The Conditional Probability Table of “Skin of the limbs (Sl)”.

	P(Sl=0)	P(Sl=1)	P(Sl=2)	P(Sl=3)
Diet =0	0.6155	0.2962	0.0808	0.0115
Diet =1	0.1818	0.3939	0.2727	0.1515
Diet =2	0	0.6	0	0.4
Diet =3	0	0	0.5	0.5

Table SS17: The Conditional Probability Table of "Sternocostal and abdominal pain (Sap)".

	P(Sap=0)	P(Sap=1)	P(Sap=2)	P(Sap=3)
Diet =0	0	0.9038	0.0962	0
Diet =1	0	0.7273	0.2424	0.0303
Diet =2	0	0.6	0.2	0.2
Diet =3	0	0	0.5	0.5

Table SS18: The Conditional Probability Table of "Chilly (Ch)".

	P(Ch=0)	P(Ch=1)	P(Ch=2)
Sl =0	0.9152	0.0788	0.0061
Sl =1	0.7849	0.1398	0.0753
Sl =2	0.5484	0.2258	0.2258
Sl =3	0.6364	0.3636	0

Table SS19: The Conditional Probability Table of "Facial features (Ff)".

	P(Ff=0)	P(Ff=1)	P(Ff=2)	P(Ff=3)	P(Ff=4)
Sl =0	0.703	0.2606	0.0242	0.0121	0
Sl =1	0.3763	0.3817	0.2043	0.0323	0
Sl =2	0.1935	0.4194	0.2903	0.0645	0.0323
Sl =3	0	0.2727	0.1818	0.4545	0.0909

Table SS20: The Conditional Probability Table of "Cardiothoracic (Ca)".

	P(Ca=0)	P(Ca=1)	P(Ca=2)
Sl =0	0.9394	0.0424	0.0182
Sl =1	0.8817	0.1075	0.0108
Sl =2	0.5806	0.2903	0.129
Sl =3	0.4545	0.0909	0.4545

Table SS21: The Conditional Probability Table of "Defecate and urine (Du)".

	P(Du=0)	P(Du=1)	P(Du=2)
Sl =0	0.7879	0.1939	0.0182
Sl =1	0.6451	0.2688	0.086
Sl =2	0.2581	0.5161	0.2258
Sl =3	0.2727	0.3636	0.3636

Table SS22: The Conditional Probability Table of "Sweating (St)".

	P(St=0)	P(St=1)	P(St=2)
Du=0	0.8756	0.1244	0
Du=1	0.8182	0.1558	0.026
Du=2	0.5	0.4091	0.0909

Table SS23: The Conditional Probability Table of “Sleep (Slp)”.

	P(Slp=0)	P(Slp=1)	P(Slp=2)
Sl =0	0.9273	0.0667	0.0061
Sl =1	0.828	0.1613	0.0108
Sl =2	0.4839	0.4839	0.0323
Sl =3	0.4545	0.3636	0.1818

Table SS24: The Conditional Probability Table of “Emotion (NEs)”.

	P(NEs=0)	P(NEs=1)	P(NEs=2)
Sl =0	0.89	0.103	0.0061
Sl =1	0.7419	0.2473	0.0108
Sl =2	0.5806	0.2903	0.129
Sl =3	0.2727	0.4545	0.2727
